# Supplementary material for: The prevalence of chronic pain in adolescents in Central Switzerland: A cross- sectional school-based study protocol
Source: PLoS One. 2024 Feb 8;19(2):e0297088. doi: 10.1371/journal.pone.0297088 (PMC10852288; doi:10.1371/journal.pone.0297088)
Supplement: S1 EthicsGer — (PDF) [file pone.0297088.s001.pdf]

EKNZ

Ethikkommission
Nordwest- und
Zentralschweiz

Präsident
Prof. Christoph Beglinger
Vizepräsidenten
Dr. Angela Frotzler
Dr. Marco Schärer

Helen Schwerdt
Department of Health, HES -SO, University of
Applied Sciences and Arts Western Switzerland
Haute École de Santé Vaud (HESAV)
Av. de Beaumont 21
1011 Lausanne

Basel, 11. Juli 2023 / LF

Verfügung der Ethikkommission Nordwest- und Zentralschweiz (EKNZ)

**Project-ID**	2023-00891
**Projekttitel**	The Prevalence of Chronic Pain and Pain Knowledge among School Children and Adolescents in Central Switzerland: A Cross-Sectional Study.
**Master-/Doktorarbeit von**	Schwerdt, Helen
**Projektleitung**	Helen Schwerdt
**Sponsor**	Haute École de Santé Vaud (HESAV-HES-SO),
**Zentren**	• Helen Schwerdt, Department of Health, HES -SO, University of Applied Sciences and Arts Western Switzerland, Lausanne

Entscheid

☒ Die Bewilligung wird erteilt
Diese Bewilligung gilt für die angekündigte Dauer der Studie, höchstens jedoch für 5 Jahre ab Datum dieser Verfügung.

☐ Die Bewilligung wird mit Auflagen erteilt
Diese Bewilligung gilt für die angekündigte Dauer der Studie, höchstens jedoch für 5 Jahre ab Datum dieser Verfügung.

☐ Die Bewilligung kann noch nicht erteilt werden

☐ Die Bewilligung wird nicht erteilt

☐ Auf das Gesuch wird nicht eingetreten

Anmerkungen / Auflagen / Bedingungen / Begründung

→ Die Bedingungen vom 09. Juni 2023 wurden erfüllt.

Klassifizierung

☒ Forschungsprojekt gemäss HFV — Kategorie: A

- ☒ Forschung mit Personen
- ☐ Weiterverwendung des biologischen Materials oder der gesundheitsbezogenen Personendaten
- ☐ mit Verstorbenen

- ☐ mit Embryonen / Föten
- ☐ mit ionisierender Strahlung

Entscheidverfahren

☐ ordentliches Verfahren ☐ vereinfachtes Verfahren ☒ Präsidialverfahren

Die Ethikkommission bestätigt, dass sie nach ICH-GCP arbeitet.

Gebühren

Betrag: CHF.-- **Tarifcode:**
Gemäss der geltenden Gebührenordnung von swissethics.

Rechtsmittelbelehrung

Gegen diesen Entscheid kann innert 30 Tagen seit dessen Zustellung beim Kantonsgericht Luzern, 4. Abteilung, Postfach 3569, 6002 Luzern, Verwaltungsgerichtsbeschwerde erhoben werden. Die Beschwerde ist im Doppel einzureichen. Sie hat einen Antrag und dessen Begründung zu enthalten.

Kopie an

- ☐ BAG
- ☒ Sponsor Haute École de Santé Vaud (HESAV-HES-SO), helen.schwerdt@hesav.ch
- ☐ Andere

Unterschrift

i.V. [signature]

Prof. Dr. med. Christoph Beglinger
Präsident

Anhang:
1. Pflichten des Sponsors/der Prüfperson oder der Projektleitung
2. Mögliche Entscheide und ihre Bedeutung
3. Eingereichte Dokumente

Anhang 1

Pflichten des Sponsors/der Projektleitung

Einreichung Dokumente: revidierte Dokumente und neue Dokumente zur Studie/zum Projekt sollen ausschliesslich über das Web-Portal BASEC eingereicht werden, auf der entsprechenden Formularseite des betreffenden Gesuches. Obsolete Dokumente sind dabei zu entfernen und Datums- und Versionsangaben entsprechend zu ergänzen. Die erfolgten Änderungen müssen im Korrekturmodus abgefasst werden und zusätzlich als ‚clean'-Version eingereicht werden. Die Studieninformationen und -einwilligungen, das Protokoll und die Amendments müssen in durchsuchbaren PDF-Dateien eingereicht werden, insbesondere müssen gescannte Dokumente eine Texterkennung durchlaufen haben (OCR). Das unterschriebene und datierte Begleitschreiben muss die Antworten auf eventuell von der EK gestellte Fragen enthalten. Revidierte Dokumente sind auch den weiteren Zulassungsbehörden zuzustellen, sofern diese involviert sind.

Anmerkung: Die zuständige Ethikkommission überprüft im Rahmen des Bewilligungsverfahrens Aufklärungsbogen und Einwilligungserklärung in einer der Amtssprachen Deutsch, Französisch oder Italienisch. Aufklärungsbogen

und Einwilligungserklärung in einer anderen Sprache werden von der Ethikkommission lediglich zur Kenntnis genommen. Für die korrekte Übersetzung ist der Sponsor oder die Projektleitung verantwortlich.

Meldepflichten: Die rechtlich bindenden Melde- resp. Bewilligungspflichten an die Ethikkommission für wesentliche Änderungen, einen vorzeitigen Studienabbruch, unerwünschte Ereignisse u.a. sind einzuhalten (Verordnungen des Bundes). Der Abschlussbericht ist spätesten ein Jahr nach Studienende der Ethikkommission einzureichen.

Registrierungspflicht: Der Sponsor muss – falls es sich um einen klinischen Versuch handelt – diesen in einem WHO-Primärregister oder im Register der Nationalen Medizinbibliothek der USA (clinicaltrials.gov) erfassen und anschliessend diese Nummer im BASEC-Portal eingeben. Die Übertragung der erforderlichen Daten in das Swiss National Clinical Trials Portal (SNCTP) kann nach Bewilligung der Ethikkommission und Zustimmung des Gesuchstellers automatisch erfolgen. Die Informationen über den klinischen Versuch sind in beiden Registern öffentlich zugänglich. Zusätzlich veröffentlicht swissethics wenige Informationen wie Titel, Projekttyp oder Leit-Ethikkommission aller durch die kantonalen Ethikkommissionen bewilligten Gesuche auf swissethics.ch (ausser Phase-I-Studien).

Anhang 2

Mögliche Entscheide und ihre Bedeutung

Die Bewilligung wird erteilt: Das Vorhaben kann gemäss bewilligtem Forschungsplan und im Rahmen der anwendbaren rechtlichen Bestimmungen durchgeführt werden. Weitere Bewilligungspflichten (Swissmedic/BAG) sind zu beachten

Die Bewilligung wird mit Auflagen erteilt: Das Vorhaben kann gemäss bewilligtem Forschungsplan gestartet werden und im Rahmen der anwendbaren rechtlichen Bestimmungen durchgeführt werden. Die Auflagen sind zu erfüllen und die Gesuchsunterlagen innert 30 Tagen entsprechend anzupassen. Die revidierten Dokumente werden nach Einreichung im Präsidialverfahren geprüft. Weitere Bewilligungspflichten (Swissmedic/BAG) sind zu beachten

Die Bewilligung kann noch nicht erteilt werden: Das Vorhaben kann noch nicht gestartet werden. Die nachfolgenden Bedingungen sind zu erfüllen bzw. die Fragen zu beantworten und die revidierten Dokumente erneut bei der Ethikkommission einzureichen. Die Ethikkommission überprüft die revidierten Dokumente und erteilt die Bewilligung, wenn die Bedingungen erfüllt bzw. die Fragen zufriedenstellend beantwortet sind.

Die Bewilligung wird nicht erteilt: Das Vorhaben kann in der vorliegenden Form nicht durchgeführt werden. Eine Neueinreichung ist möglich.

Auf das Gesuch wird nicht eingetreten: Begründung siehe vorne, z.B. nicht zuständig oder nicht bewilligungspflichtig.

Anhang 3

Eingereichte Dokumente für das Hauptzentrum

Helen Schwerdt, Department of Health, HES -SO, University of Applied Sciences and Arts Western Switzerland, Lausanne

Dokument	Kategorie	Dok.Datum	Version
explanation-changes-v2.pdf	1. Cover Letter	05/07/2023	
pi-jugendliche-14-17-hfv-d-tcm-v2.docx	3. ICF	05/07/2023	Version 2
pi-kinder-11-13-hfv-d-clean-v2.docx	3. ICF	05/07/2023	Version 2
pi-kinder-11-13-hfv-d-tcm-v2.docx	3. ICF	05/07/2023	Version 2
pi-eltern-vormund-11-14yo-hfv-d-tcm-v2.docx	3. ICF	05/07/2023	Version 2
pi-eltern-vormund-11-14yo-hfv-d-clean-v2.docx	3. ICF	05/07/2023	Version 2
pi-jugendliche-14-17-hfv-d-clean-v2.docx	3. ICF	05/07/2023	Version 2
signatures.pdf	4. Study plan	05/07/2023	Version 2
hro-research-plan-tcm-v2.docx	4. Study plan	05/07/2023	Version 2
hro-research-plan-clean-v2.docx	4. Study plan	05/07/2023	Version 2
crf-german-tcm-v2.docx	5. CRF	05/07/2023	Version 2

crf-german-clean-v2.docx	5. CRF	05/07/2023	Version 2
teacher-agreement-v2.docx	9. Agreement	05/07/2023	
research-agreement-hesav-ucd.pdf	9. Agreement	04/07/2023	
recruting-mail-v2.docx	11. Other documents for participants	05/07/2023	Version 1
recruting-mail-v2.docx	11. Other documents for participants	05/07/2023	Version
einladung-informationsveranstaltung-clean-v2.docx	11. Other documents for participants	05/07/2023	Version 2
einladung-informationsveranstaltung-tcm-v2.docx	11. Other documents for participants	05/07/2023	Version 1
