# Supplementary material for: The prevalence of chronic pain in adolescents in Central Switzerland: A cross- sectional school-based study protocol
Source: PLoS One. 2024 Feb 8;19(2):e0297088. doi: 10.1371/journal.pone.0297088 (PMC10852288; doi:10.1371/journal.pone.0297088)
Supplement: S2 EthicsGer — (DOCX) [file pone.0297088.s002.docx]

**EKNZ Ethikkommission Nordwest- und Zentralschweiz**

President

Prof. Christoph Beglinger

Vizepräsidenten

Dr. Angela Frotzler

Dr. Marco Schärer

Helen Schwerdt

Department of Health, HES -SO, University of Applied Sciences and Arts Western Switzerland Haute Ecole de Sante Vaud (HESA V)

A v. de Beaumont 21

1011 Lausanne

Basel, 11 . Juli 2023 / LF

**Order of the Ethics Committee Northwestern and Central Switzerland (EKNZ)**

Project ID: 2023-0089 1

Project title: The Prevalence of Chronic Pain and Pain Knowledge among School Children and Adolescents in Central Switzerland: A Cross-

Sectional Study.

Doctoral Thesis by Schwerdt, Helen

Project management Schwerdt, Helen

Sponsor Haute École de Santé Vaud (HESAV-HES-SO)

Centres Helen Schwerdt, Department of Health, HES -SO, University of Applied Sciences and Arts Western Switzerland, Lausanne

**Decision**

The authorisation is granted

This authorisation is valid for the announced duration of the study, but for a maximum of 5 years from the date of this order.

The permit is granted subject to conditions

This authorisation is valid for the announced duration of the study, up to a maximum of 5 years from Date of this order.

The authorisation cannot yet be granted

The authorisation shall not be granted

The request is not granted

**Remarks / Conditions / Justification**

The conditions of 09 June 2023 have been fulfilled.

**Classification**

Research project according to [Ordinance on Human Research with the Exception of Clinical Trials (HRO)](https://www.fedlex.admin.ch/eli/cc/2013/642/en)

Research with persons

Further use of the biological material or health-related personal data

with deceased

with embryos / foetuses

with ionising radiation

**Decision-making process**

Due process simplified procedure Presidential procedure

The Ethics Committee confirms that it works according to [ICH-GCP](https://ichgcp.net/de).

**Fees**

**Amount:** CHF.-- **Tariff code:**

According to the current swissethics fee schedule.

**Remedies**

**An administrative appeal against this decision may be lodged with the Cantonal Court of Lucerne, 4th Division, P.O. Box 3569, 6002 Lucerne, within 30 days of the date of service. The appeal must be submitted in duplicate. It must contain an appeal and the reasons for it.**

**Copy to**

**BAG, Federal Office of Public Health**

**Sponsor Haute École de Santé Vaud (HESAV-HES-SO)**

**helen.schwerdt@hesav.ch**

**Other**

**Signature**

Prof. Dr. med. Christoph Beglinger Präsident

**Appendix**:

l. Duties of the sponsor/investigator or project management

2. Possible decisions and their significance

3. documents submitted
