# Supplementary material for: The prevalence of chronic pain in adolescents in Central Switzerland: A cross- sectional school-based study protocol
Source: PLoS One. 2024 Feb 8;19(2):e0297088. doi: 10.1371/journal.pone.0297088 (PMC10852288; doi:10.1371/journal.pone.0297088)
Supplement: Attachment — Submitted filename: Response to Reviewers.pdf [file pone.0297088.s003.pdf]

UCD School of Public Health,
Physiotherapy and Sports Science

UCD Health Sciences Centre,
University College Dublin,
Belfield, Dublin 4, Ireland

T +353 1 716 6500
F +353 1 716 6501

Scoil na Sláinte Poiblí, na
Fisiteiripe agus Eolaíocht an
Daonra UCD

Ionad Eolaíocht Sláinte,
Belfield, Baile Átha Cliath 4, Eire

physio.therapy@ucd.ie
www.ucd.ie/physioperformsci

29th November 2023

Dear Reviewers and Editor,

Re: Manuscript PONE-D-23-25329
The Prevalence of Chronic Pain in Children and Adolescents in Central Switzerland: A Cross-Sectional School-based Study Protocol.

Thank you to the reviewers for their time and attention in reviewing this manuscript. Our responses to the reviewers' comments are detailed below. We hope they find these acceptable. New or amended text is indicated in red coloured text, as in the revised manuscript.

Reviewer #1

1) The authors assert in the introductory part that chronic pain is a prevalent manifestation of musculoskeletal disorders (MSD). Following this assertion, there appears to be no further instances in which the authors ascribe ongoing pain to MSD. It is unclear whether the authors' intention is to assess the prevalence of chronic pain specifically attributed to MSD or chronic pain in general.

We mean to estimate the prevalence and impact of chronic pain in children in the broadest sense with respect to aetiology. We have removed all references to 'musculoskeletal pain' and now use the general term 'chronic pain' throughout.
We changed the following text in the abstract: Chronic pain is ~~a common symptom of musculoskeletal disorders, leading to~~ associated with substantial personal suffering and societal costs and is a growing healthcare concern worldwide. [Abstract. Lines 26/27]

2) I am also interested in ascertaining the precise details of the information that was communicated to the school administration, as well as the individual responsible for collecting the data.

We have included the following to clarify the information that was conveyed to the school administrators "The objectives of the study and the data collection procedure were presented to the school principles to provide them with a comprehensive understanding of the study." [Recruitment. Lines 179/180]

3) I comprehend that the authors intend to employ a body chart as a means of discerning the specific locations of pain originating from various regions of the human body. However, it remains unclear if the authors intend to gather data on pain severity during periods of rest or activity.

We intend to gather a generic measure of pain severity as 'usually' perceived and experienced and does not distinguish between rest or activity. To clarify this we have added the following text: "Usual p~~P~~ain intensity by asking, "How ~~strong~~ intense the pain feels usually?", using an 11-point Numerical Rating Scale (NRS-11) (0 = no pain to 10 = the worst pain you can imagine". This question provides a

generic pain intensity and does not distinguish between pain at rest or activity (46)". [Primary outcomes. Lines 306-314]

4) Additionally, the authors state that children will be selected for recruitment based on certain inclusion criteria, although these criteria are not explicitly outlined.

The eligibility criteria are reported in the section Participants, lines 158-162, as: "Eligible participants include students aged 11 to 17 years attending classes in the second and third education cycle in Central Switzerland. The exclusion criteria for this study includes ~~children~~ adolescents who decline to consent, and ~~children~~ adolescents who cannot comprehend verbal instructions and/or read study documents in the German language." We think the eligibility (i.e. inclusion) criteria have been adequately reported; no amendments made. [Participants. Lines 158-162]

5) The protocol has weaknesses in the following aspects: The authors has not supplied any information regarding the development of the questionnaire, its suitability for young people and adolescents, the psychometric evaluation of the questionnaire, or the total time required to complete it.

In the sections 'Primary Outcomes' and 'Secondary outcomes' we report that the questionnaire instrument was developed from and based on pre-designed questionnaires from a number of cited studies from within the field. We also report their psychometric properties where data is available; and we acknowledge where it is not. We have added the following text to the 'Limitations' section: "The study outcomes are based, in part, on existing pain-related instruments used in similar studies (e.g., pain intensity, pain locations). The reliability and validity of our exact instrument has not been tested but is inferred from the available psychometric data upon which the original questionnaires are based." We hope this is acceptable. [Limitations. Lines 467- 473]

To clarify the time to complete the questionnaire we included the following information in the section Outcome measures: "Piloting the questionnaires ensured that the questions were comprehensible and took approximately ten minutes to answer." [Outcome measures. Lines 268/269]

6) It is imperative to provide information regarding the duties of any committees involved in the study, as well as specifying the individuals responsible for overseeing the study.

More information on the committees involved are given in the section Methods, lines 148-154. The paragraph has been amended to read as follows: "This study is being undertaken in part fulfilment of a Doctor of Philosophy (PhD) degree at University College Dublin (UCD) in partnership with Haute Ecole de Santé Vaud (HESAV, HES-SO). As a principal investigator (HS) leads the study, with guidance and support from an academic supervisor (KS) and a co-supervisor (GC). The study is also supported by an international collaborator with expertise (JP) and an academic with statistical expertise (CB). The study is overseen by a research study panel with an independent chairperson." [Methods. Lines 148-154]

7) Is there a limitation on the concurrent healthcare services offered to the participants during the data gathering process? In light of the findings of the study, what methods have been formulated for post-care?

This is an epidemiology study in the Swiss school system. It is taken place independent of any health care services. There is no health care provided as part of the study.

8) What are the proposed strategies for disseminating the findings or results of this study?

Our strategies for disseminating the findings from this study have been described [Funding and Dissemination. Lines 489-494]. We have added the following text: "Findings will be further disseminated via relevant clinical and academic conferences (held by the Swiss Pain Society and the European Pain Federation for example), pain-related public and patient advocacy groups, and social media. We will present our findings to the children, parents, teachers and school administrators at a post-study online evening seminar."

9) In addition, there are some minor details that should be taken into consideration. Sentences 62-63: Does this data have global applicability or is it specific to Switzerland?

The text has been amended to "Global estimates of the prevalence of chronic pain in school children from 11 to 17 years old varies from 3 to 37% (5,9,11,12)." [Introduction. Lines 70-72]

10) Please provide a more detailed explanation of the erroneous and unhelpful assumptions around pain experienced by children.

In the introduction, the following sentence is added to clarify the erroneous and unhelpful assumptions around pain: "In recent decades, it has become increasingly evident that knowledge and beliefs about pain can contribute to the duration of pain and pain-related disability in adults (16–19) and in adolescents (20-21). ~~Misconceptions and unhelpful beliefs about pain are prevalent among adults (16,20,21). "Pain is a sign of damage" and "The body needs protecting, rest and avoidance to heal." are examples of common misconceptions (16,22,23). Children acquire these concepts from their community and may also hold other misconceptions, such as age being a determinant of pain sensitivity (24).~~"Examples of common misconceptions about pain in adolescents are that pain always signifies harm, that the body needs rest and avoidance to heal, and that emotions doesn't influence pain (20,21)." [Introduction. Lines 81-114]

11) Sentence 64: What is the significance of regional differences?

We have amended the text to: "This ~~wide variation can likely~~ variability may be explained by different definitions of chronic pain, and data collection methods~~, and reginal differences~~ (9,12)." [Introduction. Lines 72/73]

13) Sentences 84-87: Kindly review again.

We are unsure of the reviewer's issue here and on reviewing the text we do not think any changes are required. However, we have swapped 'children' for 'adolescents'.

14) Given the dearth of prevalence estimates, what are the reasons for confining the investigation to Central Switzerland?

Limited financial resources restricted the scope of the study, making it more feasible to focus on a specific region rather than attempting a broader nationwide investigation. Due to resource limitations, it was more practical to conduct the study within a local area, Central Switzerland, where logistical and operational challenges could be managed more effectively.

15) Who will collect the data, how the data will be managed securely, and who will feed the data into the computers?

In the section Data collection the following paragraph clarifies the procedure: "One researcher (HS) will be present in the classroom together with the teacher to ~~will~~ administer the link to the questionnaires or reading substitute task to all ~~children~~ adolescents. This also ensures direct enquiries can be made and compliance with the study protocol. Excluded children receive help from HS and the teacher to decide on the substitute task. Participating ~~children~~ adolescents will be invited to complete questionnaires related to i) their demographics (age, gender, class grade), ii) the absence/presence of chronic pain, iii) pain characteristics and impact (those with chronic pain only), and iv) knowledge and beliefs about pain. The demographic data and the pain-related questionnaires will be completed via the cloud-based survey tool LimeSurvey.
During the data collection period ~~anonymous~~ anonymised data will be stored digitally in the LimeSurvey cloud and will be accessible ~~by~~ to the research team only. After each data collection session the ~~final adolescents is enrolled the study~~ data will be exported to ~~extracted~~ and stored in a password protected folder on the server at Haute École de Santé Vaud in Switzerland for ten years. All data will be handled in accordance with current Swiss legislation (37). [Data collection. Lines 250-264]

16) Given that the study adopts a cross-sectional design, the authors posit the elements that they perceive to be responsible for the occurrence of missing data. What measures would be implemented to ensure the participants' compliance with the study protocol?

It has been clarified that the teacher and the PI are physically present in the room with the participants to ensure compliance with the study protocol. [Data collection. Lines 252/253]

17) Please add a section that will discuss the methodology for data collection and management, with a particular focus on safeguarding personal information and ensuring confidentiality.

The section Data collection is specified as outline above. [Data collection. Lines 250-264]

18) Kindly present a section elucidating the authors' strategies to mitigate the impact of confounding variables, encompassing the utilisation of current medications.

It is not feasible to us to collect data on medication usage. We have added the text to the limitations in the section Limitations: "The scope of our study does not enable us to collect data on medication usage, the prevalence of chronic pain among parents or socio-economic status. The extent to which these variables might confound our findings is not known." [Limitations. Lines 472-474]

19) Sentence 300 need rephrasing.

"Specifically, this study will provide estimates of the severity, duration, and location of chronic pain in adolescents and the extent to which it impacts on their physical, psychological and social functioning. ~~the most common locations of pain, their duration, and intensity among Swiss school children, as well as the consequences of chronic pain on their daily lives. Which is fundamental in diagnosing, treating, and preventing chronic pain in children."~~ [Limitations. Lines 432-434]

<u>Reviewer #2</u>

1) (..) As a suggestion, authors could also add socioeconomic level data to somehow consider the recruitment bias.

Thank you for the suggestion. Given that children may not be able to accurately report this information, and considering the potential burden it would impose on parents, the decision was

made not to attempt to collect socioeconomic data. We have added the text to the limitations in the section Limitations: "The scope of our study does not enable us to collect data on medication usage, the prevalence of chronic pain among parents or socio-economic status. The extent to which these variables might confound our findings is not known." [Limitations. Lines 472-474]

General requirements:

1. Please ensure that your manuscript meets PLOS ONE's style requirements, including those for file naming. The PLOS ONE style templates can be found at https://journals.plos.org/plosone/s/file?id=wjVg/PLOSOne_formatting_sample_main_body.pdf and https://journals.plos.org/plosone/s/file?id=ba62/PLOSOne_formatting_sample_title_authors_affiliations.pdf
The name of the document is adapted to the guideline.

2. We note that you have stated that you will provide repository information for your data at acceptance. Should your manuscript be accepted for publication, we will hold it until you provide the relevant accession numbers or DOIs necessary to access your data. If you wish to make changes to your Data Availability statement, please describe these changes in your cover letter and we will update your Data Availability statement to reflect the information you provide.

"On completion of the study we will make our anonymised data available on the Open Science Framework." [Methods. Lines 168] DOI: 10.17605/OSF.IO/Z3QF6.

3. Ethics statement only appears at the end of the manuscript:
Your ethics statement should only appear in the Methods section of your manuscript. If your ethics statement is written in any section besides the Methods, please move it to the Methods section and delete it from any other section. Please ensure that your ethics statement is included in your manuscript, as the ethics statement entered into the online submission form will not be published alongside your manuscript.
The ethic statement is replaced to the methods [Line 223-232] and deleted in the Ethic, Funding and Dissemination Section. This section is renamed to Funding and Dissemination. [Funding and Dissemination. Lines 485]

Additional changes:
1) According to the WHO's definition of age groups, pupils aged 11-17 years are considered adolescents. Therefore, the term 'children' has been revised to 'pupils and adolescents' throughout the manuscript. The title has also been revised: "The Prevalence of Chronic Pain ~~in Children and~~ in Adolescents in Central Switzerland: A Cross- Sectional School-based Study Protocol." [Title. Lines 1-3]

2) To clarify the aims in the abstract we changed the following text: "This study aims to estimate the prevalence, characteristics, and impact of chronic pain~~, investigate its characteristics, and impact~~ and explore ~~children's~~ adolescents' knowledge and beliefs about pain." [Abstract. Lines 30-32]

3) We have edited the following text for clarification:
- "High quality ~~methods of~~ prevalence ~~data collection~~ studies have been recommended in order to provide more accurate estimates of the prevalence of chronic pain in ~~children~~ adolescents (9,12,13). [Introduction. Lines 62-64]
- "The latest estimat~~e~~ion ~~revealed~~ suggested a nationwide Swiss prevalence of 3% of chronic pain in adolescents ~~children~~. [Introduction. Lines 78/79]

4) The description of the PROMIS PPI Questionnaire has been adapted for clarification. “The impact of their pain using the Patient Reported Outcome Measures Information System - Pediatric Pain Interference Scale (PROMIS-PPIS) short version in German (51). PROMIS-PPI assesses self-reported impairments in physical, psychological, and social functioning.” [Primary outcomes. Lines 334/348]

5) We asked twice about chronic pain locations. To clarify the specific location referred to in the first question, we rephrased the following sentence in the primary outcome section.:
For the first body chart, the child will be asked to mark the region in which they feel their most bothersome chronic pain. [Primary outcomes. Line 300]

6) To clarify the funding scheme we added the following text: “This study is funded by a Swiss – Irish Joint Doctoral Programme in Health Sciences (Health Sciences PhD) scheme.” [Funding and Dissemination. Lines 515/516]

Thank you for re-reviewing our protocol for publication in PLOS ONE.

Yours sincerely,

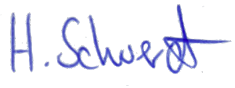

Helen Schwerdt, PhD cand.
Mail: Helen.schwerdt@ucdconnect.ie
Tel. : +41 78 208 15 33
