# Supplementary material for: The prevalence of chronic pain in adolescents in Central Switzerland: A cross- sectional school-based study protocol
Source: PLoS One. 2024 Feb 8;19(2):e0297088. doi: 10.1371/journal.pone.0297088 (PMC10852288; doi:10.1371/journal.pone.0297088)
Supplement: Attachment — Submitted filename: Response to Reviewers.docx [file pone.0297088.s004.docx]

18^th^ December 2023

Dear Reviewers and Editor,

Re: Manuscript PONE-D-23-25399R1

The Prevalence of Chronic Pain in Adolescents in Central Switzerland: A Cross-Sectional School-based Study Protocol.

Thank you to the reviewers for their time and attention in reviewing this manuscript again. Our responses to the reviewers’ comments are detailed below. We hope they find these acceptable. New or amended text is indicated in red coloured text, as in the revised manuscript.

4. Have the authors described where all data underlying the findings will be made available when the study is complete?

The PLOS Data policy requires authors to make all data underlying the findings described in their manuscript fully available without restriction, with rare exception, at the time of publication. The data should be provided as part of the manuscript or its supporting information or deposited to a public repository. For example, in addition to summary statistics, the data points behind means, medians and variance measures should be available. If there are restrictions on publicly sharing data—e.g. participant privacy or use of data from a third party—those must be specified.

Reviewer #1: No

As a protocol, there are no data associated with this article. On completion of the study we will make our anonymised data available without restriction on the Open Science Framework. We report this as: “On completion of the study we will make our anonymised data available on the Open Science Framework (35).” [Data collection. Lines 188 - 190]

Reviewer #1: Dear authors,

The revised manuscript has successfully addressed the majority of the issues that were raised. Nevertheless, it is necessary to revise the work for the English language. Please consider the following concerns during the process of revision:

• Correct spelling errors in sentences 61, 125, and so on.

• As an individual residing outside of the European Union, my knowledge of the enrolment status of all adolescents in educational institutions is limited. The primary objective of the authors is to assess the prevalence of pain among adolescents and not necessarily school going adolescents. The data for this study is exclusively collected from participants who attend school. The extent to which school-related pressures contribute to the perception of pain is uncertain. Either present it as a limitation or adjust the subject matter accordingly.

• Sentence 106–107: Given that the timing of this revision is far beyond September 2023, it is advisable to revise the tense where appropriate.

1. The manuscript has been corrected for English language as follows:

Sentence 61: Global estimates of the prevalence of chronic pain in adolescents from aged 11 to 17 years old var~~i~~y from 3 to 37% (5,9,11,12). [Introduction. Lines 60-62]

Sentence 125: The objectives of the study and the data collection procedure were presented to the school ~~principles~~ principals to provide them with a comprehensive understanding of the study. [Recruitment. Lines 125 - 127]

We have corrected the following paragraphs:

“~~The following~~ ~~e~~Examples of common misconceptions about pain in adolescents are that pain always signifies harm, that the body needs rest and avoidance to heal, and that emotions do not influence pain (20,21). These conceptions are connected to expectations, which in turn shape the experience of pain (22–26). In addition to increased pain, expectations can also lead to avoidance behaviour (27–30). These ~~detrimental~~ beliefs are at odds with ~~a~~ contemporary understanding of pain science. While the majority of pain belief research has focused on adults, limited studies have identified the existence of some inaccurate and unhelpful beliefs in adolescents ~~children~~ (28,31–33). Further data is required to expand our understanding of conceptions of chronic pain in adolescents (34).” [Introduction. Lines 73 - 81]

“Currently, 4 ~~four~~ schools with about 400 possible adolescents ~~children~~ have provisionally confirmed their participation.” [Setting. Lines 122 -123]

“School principals, teachers, parents/legal guardians and pupils will be able to contact the ~~principal investigator (~~PI~~)~~ (HS) who will discuss the study and ask any questions via mail, telephone or an information session.” [Recruitment. Lines 145 - 147]

“All pupils receive the same online link. As an additional verification of willingness to participate, adolescents can decide to complete ~~The first step will be the decision between~~ the survey ~~and the participation in~~ or a reading substitute task. If ~~the~~ a participant ~~will~~ chooses the reading substitute task, a text previously chosen by the teacher will appear on the screen instead of the survey.” [Recruitment. Lines 158 - 161]

“~~One Researcher~~ The PI (HS) will be present in the classroom together with the teacher to administer the link to the questionnaires or reading substitute task to all adolescents.” [Data collection. Lines 173 - 174]

The following sentence has been moved from Outcome measures to the section Data collection: “Piloting the questionnaires ensured that the questions were comprehensible and took approximately ten minutes to answer.” [Data collection. Lines 181 - 182]

“For the purpose of this study, we operationally defined the presence of chronic pain as the self-reported experience of recurrent or persistent pain for three or more ~~than three~~ months and a frequency of at least weekly [38–42]. This definition was chosen because it is the most commonly used and can therefore be compared with other studies from ~~in~~ the field [9,12].” [Primary outcomes. Lines 199 - 203]

“The presence/absence of chronic pain will be determined by participants’ self-reported responses to the following two questions:” [Primary outcomes. Lines 204 - 205]

“The chronic pain sites have a score range from 0 to 12~~. The coding is according to a list~~ based on axis I of the IASP Classification of Chronic Pain [43] and has been used in previous studies investigating pain in adolescents [5,40,44]. Its reliability and validity are unknown.” [Primary outcomes. Lines 219 - 222]

“The NRS-11 has good convergent validity~~, as it~~ showing moderate to high correlations with other pain intensity scales [46]” [Primary outcomes. Lines 230 - 231]

“Threshold values have been identified. Average scores of 3, 6, and 8 on the NRS-11 correspond~~ed~~ to mild, moderate, and severe pain levels, respectively [46]. The NRS-11 has been used in a number of pain-related pediatric epidemiological studies ~~to ask for the chronic pain intensity~~ [40–42].” [Primary outcomes. Lines 236 - 239]

“It has been used in numerous studies in adolescents [41,47–49] and will facilitate comparisons of ~~be used in order to be able to compare the~~ results.” [Primary outcomes. Lines 243 - 244]

“The construct validity ~~of~~ was confirmed~~, as evidenced~~ by good goodness-of-fit indexes.” [Primary outcomes. Lines 266 - 267]

“Next the relationship between chronic pain and ~~total concept of pain (~~COPI-GER score~~)~~ as well as individual items in the child’s COPI-GER, independent of age and gender, will be explored with logistic regression models. Results will be expressed as odds ratios.” [Statistical Analysis Plan. Lines 284 - 287]

“The calculation is based ~~of~~ on a population size of 86424 adolescents ~~children~~ between 11 and 17 years in central part of Switzerland (canton Luzern, Aargau, Schwyz and Zug) in 2021.” [Sample Size. Lines 310 - 312]

This study will attempt to provide a more precise estimate of the prevalence, characteristics, and impact of chronic pain in adolescents aged between 11 and 17 years in Central Switzerland compared to previous research [15]. ~~The results will present the characteristics of chronic pain and its impact on adolescents.~~

~~As highlighted in the introduction, it is crucial to identify and address any~~ Unhelpful beliefs and misconceptions about pain~~, as they can~~ may contribute to maladaptive behaviours and the development of chronic pain. ~~By detecting~~ Identifying specific misconceptions about pain~~, this study will~~ could inform future studies that design interventions to target and rectify these misconceptions and better ~~potential interventions aimed at~~ align adolescents’ understanding of pain with contemporary pain science. Such understanding may ultimately contribute to the prevention and treatment of chronic pain in adolescents ~~children~~. Future projects can utilize the findings to design interventions that target and rectify these misconceptions. [Discussion. Lines 318 - 330]

“The potential limitations of self-reporting when using PROMs to measure our outcomes of interest are acknowledged ~~considered~~ [60.]” [Limitations. Lines 339 - 340]

“To reduce this~~e~~ bias, a reference point will be discussed with the teacher beforehand to help the adolescents accurately estimate the duration of the past three months.” [Limitations. Lines 341 - 343]

2. The study was declared as a school-based study in the title and in the methods. In order to specify the influence of school pressure on the results, which was not surveyed, the text has been adjusted in the ‘Limitations’ section as follows: “The scope of our study does not enable us to collect data on medication usage, the influence of any perceived school-related pressures, the prevalence of chronic pain among parents or socio-economic status. The extent to which these variables might confound our findings is not known.” [Limitations. Lines 349 - 352]

We also have adapted the following text in the abstract: “This is an observational cohort study of school-going adolescents ~~pupils~~ aged 11 to 17 years in Central Switzerland. The study will estimate the point prevalence, characteristics (location, intensity, frequency, duration) and impact (PROMIS Pediatric Short Form v2.0 – Pain Interference Scale, PPIS) of chronic pain in school-going adolescents.” [Abstract. Lines 32 - 36]

3. Sentence 108-109 is in the manuscript. As the data collection is currently ongoing, we wrote the manuscript in future tense.

We have adapted the following text in line with progress:

“Data collection started ~~will commence~~ in September 2023 and will last for approximately five months.” [Study design. Lines 108 - 109]

“~~Timeline Recruitment has stopped for the time being as the required number has been reached. Responses from further schools are still pending. With the refusal of schools or too fewer participating adolescents in the schools, there will be recruiting again at any time.~~ Preparations for data collection and information events took ~~will take~~ place in August and ~~early~~ September 2023. ~~The first~~ Data collection ~~will take place~~ commenced in September 2023 and is expected to be completed in ~~to~~ January 2024 ~~on days to be determined~~. Data analysis ~~and possible publications~~ will begin on ~~take place in the following months after the~~ completion of the data collection.” [Timeline. Lines 314 - 316]

Thank you for re-reviewing our protocol for publication in PLOS ONE.

Yours sincerely,

Helen Schwerdt, PhD cand.

Mail: [Helen.schwerdt@ucdconnect.ie](mailto:Helen.schwerdt@ucdconnect.ie)

Tel. : +41 78 208 15 33
